# Melancholia Puerperalis Atonicta

**Published:** 1845-01

**Authors:** 


*Melancholia Puerperalis Attonita.*—A female in her first confinement had the misfortune to *overlie* her four days old infant, and in addition to her own distress, was scolded unmercifully by her husband, a violent man. The change that came over the poor woman was so great and sudden, that on my entrance into the room I saw death in her features. Her consciousness was intact, she had no fever; the vegetative functions appeared all to be unaffected; but the expression of the countenance was sorrowful, suffering, and yet indifferent—indescribable, but which once seen can never be forgotten. My unfavourable prognosis was not believed: the patient was comfortable, and they sent for another physician, who prescribed antiphlogistic means, apprehensive of puerperal fever, and then, when the powers of life began suddenly to fail, cordial and stimulating remedies; but all in vain; in three days the patient was a corpse.

This was no case of puerperal mania, nor yet of puerperal typhus; the cause of the mischief, as I apprehend it, lay in a kind of paralysis of the sensorium commune; not material, but mental, and striking at the very roots of life. I saw that we were without means of dealing with so serious a mental impression as had here been made. Although there is little in a name, yet I have ventured to propose one for this form of mental disease; or if the reader prefers it he may say that the patient died of a broken heart.—*Ib., from Dr. Lion, in Casper's Wochenschrift.*

